# Pharmacodynamic evaluation of piperacillin/tazobactam versus meropenem against extended-spectrum β-lactamase-producing and non-producing *Escherichia coli* clinical isolates in a hollow-fibre infection model

**DOI:** 10.1093/jac/dkac186

**Published:** 2022-06-20

**Authors:** Kamrul Islam, Fekade B Sime, Steven C Wallis, Michelle J Bauer, Brian M Forde, Patrick Harris, Tahmina Shirin, Zakir H Habib, Meerjady S Flora, Jason A Roberts

**Affiliations:** University of Queensland Centre for Clinical Research, Faculty of Medicine, The University of Queensland, Brisbane, Australia; University of Queensland Centre for Clinical Research, Faculty of Medicine, The University of Queensland, Brisbane, Australia; University of Queensland Centre for Clinical Research, Faculty of Medicine, The University of Queensland, Brisbane, Australia; University of Queensland Centre for Clinical Research, Faculty of Medicine, The University of Queensland, Brisbane, Australia; University of Queensland Centre for Clinical Research, Faculty of Medicine, The University of Queensland, Brisbane, Australia; University of Queensland Centre for Clinical Research, Faculty of Medicine, The University of Queensland, Brisbane, Australia; Herston Infectious Diseases Institute (HeIDI), Metro North Health, Brisbane, Australia; Institute of Epidemiology, Disease Control and Research (IEDCR), Mohakhali, Dhaka, Bangladesh; Institute of Epidemiology, Disease Control and Research (IEDCR), Mohakhali, Dhaka, Bangladesh; Directorate General of Health Services, Mohakhali, Dhaka, Bangladesh; University of Queensland Centre for Clinical Research, Faculty of Medicine, The University of Queensland, Brisbane, Australia; Herston Infectious Diseases Institute (HeIDI), Metro North Health, Brisbane, Australia; Departments of Pharmacy and Intensive Care Medicine, Royal Brisbane and Women’s Hospital, Brisbane, Australia; Division of Anaesthesiology Critical Care Emergency and Pain Medicine, Nîmes University Hospital, University of Montpellier, Nîmes, France

## Abstract

**Background:**

Urosepsis caused by extended-spectrum β-lactamase (ESBL)-producing *Escherichia coli* is increasing worldwide. Carbapenems are commonly recommended for the treatment of ESBL infections; however, to minimize the emergence of carbapenem resistance, interest in alternative treatments has heightened.

**Objectives:**

This study compared pharmacodynamics of piperacillin/tazobactam versus meropenem against ESBL-producing and non-producing *E. coli* clinical isolates.

**Methods:**

*E. coli* isolates, obtained from national reference laboratory in Bangladesh, were characterized by phenotypic tests, WGS, susceptibility tests and mutant frequency analysis. Three ESBL-producing and two non-producing *E. coli* were exposed to piperacillin/tazobactam (4.5 g, every 6 h and every 8 h, 30 min infusion) and meropenem (1 g, every 8 h, 30 min infusion) in a hollow-fibre infection model over 7 days.

**Results:**

Piperacillin/tazobactam regimens attained ∼4–5 log_10_ cfu/mL bacterial killing within 24 h and prevented resistance emergence over the experiment against ESBL-producing and non-producing *E. coli*. However, compared with 8 hourly meropenem, the 6 hourly piperacillin/tazobactam attained ∼1 log_10_ lower bacterial kill against one of three ESBL-producing *E. coli* (CTAP#173) but comparable killing for the other two ESBL-producing (CTAP#168 and CTAP#169) and two non-producing *E. coli* (CTAP#179 and CTAP#180). The 6 hourly piperacillin/tazobactam regimen attained ∼1 log_10_ greater bacterial kill compared with the 8 hourly regimen against CTAP#168 and CTAP#179 at 24 h.

**Conclusions:**

Our study suggests piperacillin/tazobactam may be a potential alternative to carbapenems to treat urosepsis caused by ESBL-producing *E. coli*, although clinical trials with robust design are needed to confirm non-inferiority of outcome.

## Introduction

Urinary tract infections (UTIs) are among the most frequent bacterial infections confronted by clinicians worldwide and represent a huge burden on the healthcare system due to a high likelihood of recurrence and increasing antibiotic resistance among uropathogens.^1^ Sepsis caused by UTIs is urosepsis, a systemic response triggered by an infection originating in the urogenital system.^2^ It is a common cause of ICU admission.^3^ The rate of urosepsis among all sepsis cases is approximately 31% and could potentially progress to severe sepsis or septic shock, which is associated with high morbidity and mortality.^4^

*Escherichia coli* is the most common bacterial species causing urosepsis^5^ and commonly carries extended-spectrum β-lactamase enzymes (ESBLs). ESBLs are a class of β-lactamases that inactivate most penicillins, oxyiminocephalosporins and aztreonam, but not carbapenems.^6^ Most ESBLs can be divided into TEM, SHV and CTX-M types.^7^ The majority of TEM or SHV evolved from parent enzymes such as TEM-1, TEM-2 and SHV-1 through point mutations.^8^ CTX-M types are clustered into five groups,^9^ with CTX-M-15 having become the predominant variant of CTX-M-1 cluster worldwide.^10^ The prevalence of ESBL-producing *E. coli* has reportedly increased in multiple countries.^11–14^ The rise of UTIs caused by ESBL-producing *E. coli* is alarming because of the limited treatment options,^7^ associated high mortality, prolonged hospital stays and elevated healthcare costs compared with infections due to non-ESBL-producing *E. coli*.^15,16^

Carbapenems are recommended for the treatment of pyelonephritis or complicated UTI caused by ESBL pathogens.^17^ However, with increasing prevalence of ESBL-producing *E. coli* in UTIs, overuse of carbapenems may further select carbapenem resistance.^18^ Meropenem consumption is strongly associated with resistance in *E. coli*.^19^ Therefore, there is an urgent need to identify suitable alternative antibiotics to carbapenems, which reduce selection pressure. β-lactam/β-lactamase inhibitor combination drugs such as piperacillin/tazobactam are considered as promising alternative since many ESBL-producing Enterobacterales are susceptible to them.^20^ This is partly attributable to tazobactam’s ability to inhibit enzymatic degradation of piperacillin by ESBLs thereby circumventing an important mechanism of antibiotic resistance.^21^ However, reduced piperacillin/tazobactam activity has also been reported, perhaps by other mechanisms of resistance, since genes expressing ESBLs are often located in the same plasmid that carry other genes expressing different resistance mechanisms,^22^ porin mutations or efflux pump.^21^

Clinical studies of the efficacy of piperacillin/tazobactam against ESBL infections have given conflicting results. Meropenem versus piperacillin/tazobactam for definitive treatment of bloodstream infections due to ceftriaxone-non-susceptible *E. coli* and *Klebsiella* spp. (the MERINO) randomized controlled trial (RCT)^23^ found that piperacillin/tazobactam was not non-inferior for 30 day mortality when compared with meropenem in treating bloodstream infection due to ceftriaxone-non-susceptible *E. coli* or *Klebsiella* spp. Nevertheless, this result may not be extrapolatable to all infection sources, particularly urinary sepsis caused by ESBL-producing *E. coli.* A subgroup analysis in the MERINO trial reported a lower mortality difference between urinary versus non-urinary (6.9% versus 18.8%) infections for piperacillin/tazobactam.^23^ Another RCT investigating ESBL-UTIs did not find a difference between piperacillin/tazobactam and ertapenem in mortality at 28 days.^24^ However, these RCTs^23,24^ investigated only a set of bacteria that produces ESBLs. At present, limited mechanistic microbiological studies are available evaluating the comparative pharmacodynamics of piperacillin/tazobactam versus meropenem against ESBL-producing and non-producing *E. coli*.

To address this research gap, we compared the pharmacodynamic activity of piperacillin/tazobactam versus meropenem against ESBL-producing and non-ESBL-producing *E. coli* using a susceptible MIC distribution of isolates in a dynamic *in vitro* hollow-fibre infection model (HFIM), over 7 days.

## Materials and methods

### Antimicrobial agents, susceptibility testing, phenotypic and molecular characterization of bacterial isolates

Five *E. coli* clinical isolates (CTAP#168, CTAP#169, CTAP#173, CTAP#179 and CTAP#180) were obtained from the Institute of Epidemiology, Disease Control and Research, Bangladesh, which serves as a national reference laboratory for antimicrobial resistance surveillance. This surveillance was initially supported by the U.S. CDC, and later the WHO.^25^ Bacterial stocks were prepared in CAMHB (BD, Becton, Sparks, MD, USA) containing 20% glycerol v/v and stored immediately at –80°C.

Analytical standard piperacillin (product: P1774, CAS: 59703-84-3, lot: EVOEN-PJ, Tokyo Chemical Industry Co., Ltd, Japan), tazobactam (product: 429808, lot: FCB009363, Flurochem, UK) and meropenem (product: M2279, CAS: 119478-56-7, lot: YCY8L-BF, Tokyo Chemical Industry Co., Ltd, Japan) were used to prepare stock solutions in Milli-Q water, filtered using 0.22 μm polyvinylidene fluoride syringe filter, and immediately stored at –80°C. These antibiotic stocks were thawed prior to each experiment, and used for susceptibility testing, and to prepare drug-containing agar plates. For dosing in HFIM, piperacillin/tazobactam and meropenem stock solutions were prepared from piperacillin/tazobactam (PipTAZ, 4 g/0.5 g, AFT pharmaceuticals Ltd, Australia) and meropenem (Meropenem, 1000 mg, Fresenius Kabi Pty Ltd, Australia) IV clinical formulations, and immediately stored at –80°C. The MICs of piperacillin/tazobactam and meropenem for *E. coli* were determined by broth microdilution in four replicates according to EUCAST and CLSI guidelines^26^ with EUCAST clinical breakpoints used to define antibiotic susceptibility and resistance.^27^

Phenotypic ESBL production was confirmed using a combination disc testing with cefotaxime and ceftazidime with and without clavulanic acid. WGS was performed using Illumina MiniSeq, High Output Reagent Cartridge (300 cycles) paired ends, according to the manufacturer’s instructions at the University of Queensland Centre for Clinical Research (UQCCR), Brisbane, Australia. Genomic analysis was conducted using a custom, in-house-developed, microbial genomic analysis pipeline (https://github.com/FordeGenomics/SnapperRocks) (detailed methods can be found in the Supplementary Methods, available as Supplementary data at *JAC* Online). WGS data have been submitted to NCBI under Bioproject accession no. PRJNA762607. Raw sequence read data have been deposited to the Sequence Read Archive (see Table S1).

### Mutant frequency experiments

For each *E. coli*, a 20 mL culture with an initial inoculum of 10^2^ cfu/mL was incubated in CAMHB at 37°C for 24 h. Quantitative culture was performed on samples taken at 24 h using drug-free and drug-containing CAMHA (16 and 32 mg/L of piperacillin with fixed 4 mg/L of tazobactam, and 8 and 16 mg/L of meropenem). The ratio of the concentration of bacterial sub-populations (cfu/mL) that grow on the piperacillin/tazobactam-containing agar to that of the total bacterial population that grow on drug-free agar was determined as mutant frequency of the isolates.^28^

### In vitro dynamic HFIM

The *in vitro* HFIM set-up used in this study has been described elsewhere.^29^ Cellulosic cartridges (catalogue C3008, FiberCell Systems, Inc., Frederick, MD, USA) were used in all experiments. Piperacillin and meropenem clearances were simulated using peristaltic pumps (Masterflex^®^ L/S^™^ pump, USA). Automated syringe pump (New Era, model NE-1800) was used to infuse piperacillin/tazobactam and meropenem directly into the central compartment. Three ESBL-producing and two non-producing *E. coli* (CTAP#168, CTAP#169, CTAP#173, CTAP#179 and CTAP#180) were used in the HFIM (Table 1). Non-ESBL-producing *E. coli* isolates were included in the study as a basis for comparison with the ESBL-producing *E. coli* isolates. For each study, *E. coli* isolates were suspended in 24 mL of CAMHB and incubated at 37°C with continuous agitation in a shaking water bath for a particular duration (based on growth curve analysis) to obtain an initial bacterial inoculum of ∼10^7^ cfu/mL, as noted previously.^29^ Quantitative cultures were performed on bacterial samples (1 mL) withdrawn from the extra-capillary space of the hollow-fibre cartridge at 0, 2, 4, 6, 8, 10, 24, 28, 34, 48, 72, 96, 120, 144 and 168 h. To reduce antibiotic carryover, samples were washed twice, centrifuged at 3500 **g** for 5 min and resuspended in sterile PBS. An aliquot of 100 μL of properly diluted bacterial suspension was manually plated on drug-free CAMHA and CAMHA containing piperacillin/tazobactam at 32 mg/L piperacillin with fixed 4 mg/L of tazobactam and 16 mg/L of meropenem. The lower limit of quantification (LLOQ) was 2 log cfu/mL (i.e. counts less than 10 colonies per CAMHA were not considered). The MICs for the isolates were determined at 168 h. The bactericidal activity was defined as reduction in bacterial concentration by >3 log_10_ cfu/mL from the baseline inoculum.^30^

**Table 1.** Characteristics of bacterial isolates tested in the HFIM experiments

*E. coli* isolates	MIC to TZP (mg/L)	MIC to MEM (mg/L)	ESBL phenotypic test	Antimicrobial resistance genes	MLST
CTAP#168	4	0.0156	positive	*acrF*, ***bla*_CTX-M-15_**, *bla*_EC_, *emrD*, *mdtM*, *qnrS1*	2521
CTAP#169	8	0.0624	positive	*aac(3)-IIe*, *aadA5*, *aac(6ʹ)-Ib-cr5*, *acrF*, ***bla*_CTX-M-15_**, *bla*_EC_, *bla*_OXA-1_, *catB3*, *dfrA17, emrD*, *mdtM*, *mph(A)*, *sul1*	131
CTAP#173	2	0.0312	positive	*aph(3'ʹ)-Ib*, *aph(6)-Id*, *acrF*, ***bla*_CTX-M-15_**, *bla*_EC_, *bla*_TEM-1_, *dfrA14, emrD*, *mdtM*, *mph(A)*, *sul2*, *tet(B)*	421
CTAP#179	4	0.0156	negative	*aadA2*, *acrF*, *bla*_EC_, *catA1*, *dfrA12, emrD*, *erm(B)*, *mph(A)*, *mdtM*, *qepA4*, *sul1*, *tet(B)*	38
CTAP#180	2	0.0312	negative	*acrF*, *bla*_EC_, *emrD*, *mdtM*	38

MLST, multilocus sequence typing; TZP, piperacillin/tazobactam; MEM, meropenem. EUCAST TZP clinical breakpoint for *E. coli*: susceptible, ≤8 mg/L; resistant, >8 mg/L.^27^ EUCAST MEM clinical breakpoint for *E. coli*: susceptible, ≤2 mg/L; resistant, >8 mg/L.^27^

The specific ESBL enzyme present in isolates used for this study is highlighted in bold.

Urosepsis is a systemic response due to an infection originating from the urinary tract; the plasma concentration–time profiles of the selected regimens were simulated in the HFIM. The free piperacillin and meropenem plasma concentration–time profiles with median CL_CR_ (100 mL/min) were simulated.^31^ The simulated clearance and half-life were 11 L/h and 1.10 h, respectively, for piperacillin.^32^ For meropenem, the simulated clearance and half-life were 9.38 L/h and 2.22 h, respectively.^33^ The simulated dosing regimens were 4.5 g piperacillin/tazobactam, every 6 h and every 8 h, given as 30 min infusion, and 1 g meropenem, every 8 h, administered as 30 min infusion. An untreated control arm was included and run simultaneously following similar conditions as the treatment arm for each experiment. Pharmacokinetic samples (1 mL) were taken from the outflow of the central reservoir of the HFIM at the following timepoints 0.5, 1, 2, 4, 5.83, 7.83, 23.83, 24.5, 25, 28, 29.83, 31.83, 47.83, 48.5, 49, 52, 53.83, 55.83, 71.83, 72.5, 73, 76, 77.83, 79.83, 143.83, 144.5, 145, 148, 149.83, 151.83 and 168 h. All samples were collected into cryovials and stored immediately at –80°C until analysis.

### Piperacillin/tazobactam and meropenem assay

Piperacillin and tazobactam concentrations in CAMHB were measured using a validated ultra-high performance liquid chromatography with tandem mass spectrometry (UHPLC–MS/MS) method on a Nexera liquid chromatograph connected to a 8030+ triple quadrupole mass spectrometer (Shimadzu, Kyoto, Japan). Meropenem concentrations in CAMHB were measured by an UHPLC–photo diode array (UHPLC–PDA) method on a Nexera2 liquid chromatograph connected to a SPD-M30 PDA detector (Shimadzu, Kyoto, Japan). The precision was within 3.3% and the accuracy was within 1.2% at piperacillin concentrations of 2.61, 21.8, 69.6 and 174 mg/L. For tazobactam, the precision was within 6.4% and the accuracy was within –3.3% at 0.326, 2.72, 8.70 and 21.8 mg/L of tazobactam. For the tested meropenem concentrations of 1.5, 25 and 80 mg/L in CAMHB, the precision was within 3.8% and the accuracy was within –6.4%. Samples were analysed in batches along with calibrators and quality controls and results were subject to pre-established batch acceptance criteria^34^ (see detailed assay methods in the Supplementary data).

## Results

### Phenotypic, WGS, susceptibility and mutant frequency analysis

Phenotypes, resistance profiles and MICs of piperacillin/tazobactam and meropenem for ESBL-producing and non-ESBL-producing *E. coli* isolates are presented in Table 1. The mutant frequency of CTAP#168, CTAP#169, CTAP#173, CTAP#179 and CTAP#180 at piperacillin concentration of 16 mg/L with fixed 4 mg/L of tazobactam were <1.30 × 10^–10^, 1 × 10^–8^, <1 × 10^–11^, <1.70 × 10^–10^ and <2.30 × 10^–11^, and piperacillin concentration of 32 mg/L with fixed 4 mg/L of tazobactam were <1.30 × 10^–10^, <1.10 × 10^–10^, <1 × 10^–11^, <1.70 × 10^–10^ and <2.30 × 10^–11^, respectively. The mutant frequency of CTAP#168, CTAP#169, CTAP#173, CTAP#179 and CTAP#180 with meropenem at concentrations of 8 and 16 mg/L were <1.30 × 10^–10^, <1.10 × 10^–10^, <1 × 10^–11^, <1.70 × 10^–10^ and <2.30 × 10^–11^, respectively.

### HFIM experiments

#### Pharmacokinetic profile

Pharmacokinetic profiles of piperacillin/tazobactam and meropenem against ESBL-producing and non-producing *E. coli* isolates in the HFIM experiments are presented in Figure S1. Simulated piperacillin/tazobactam and meropenem dosing regimens in HFIM and predicted versus observed pharmacokinetic parameters against ESBL-producing and non-producing *E. coli* are reported in Table S2. Comparison between piperacillin, tazobactam and meropenem concentrations for a dosing interval of the Day 3 and Day 7 in the HFIM experiments is presented in Figure S2.

#### Effect of piperacillin/tazobactam and meropenem on bacterial killing

**Figure 1.**
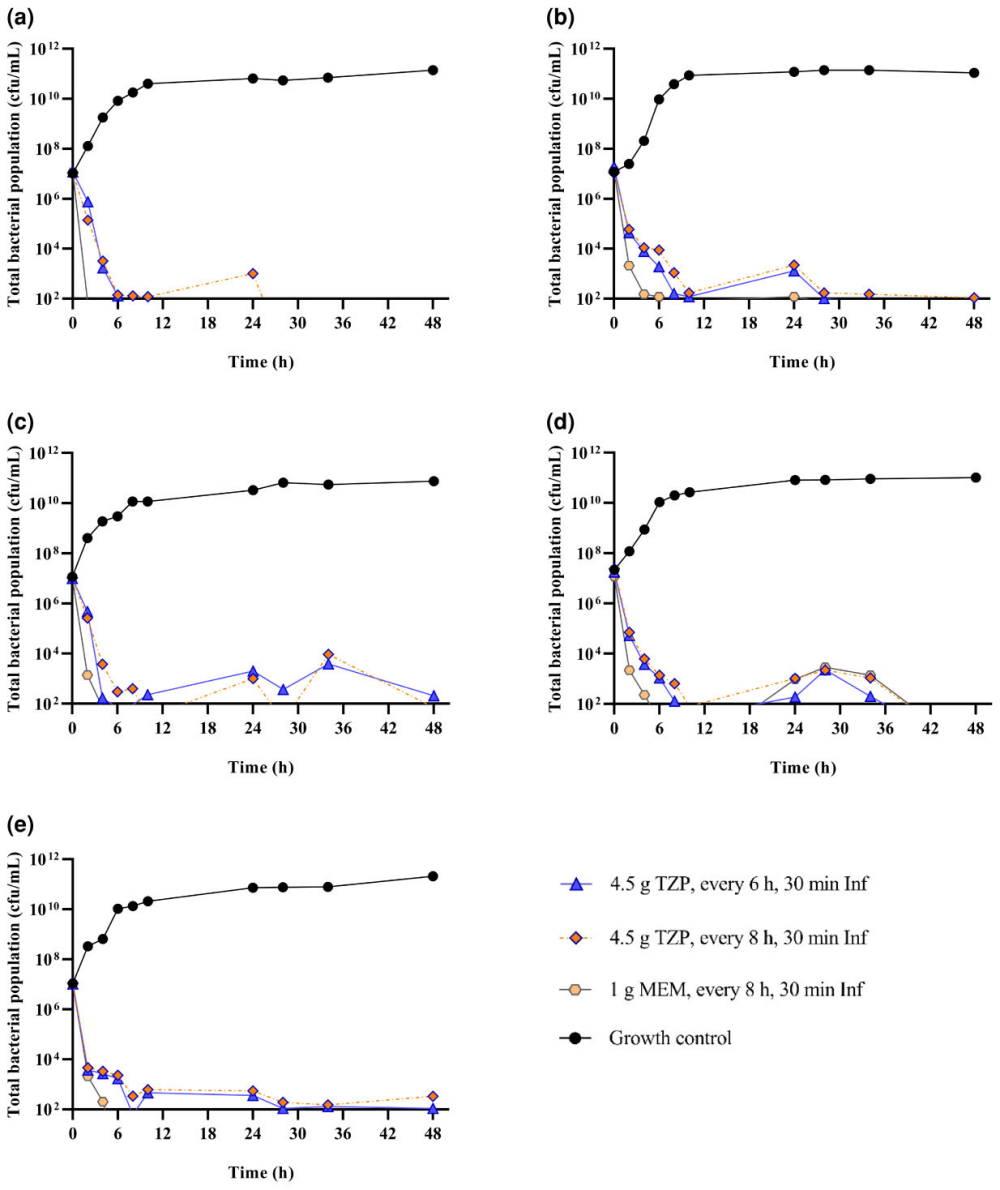
Change in total bacterial population of *E. coli* investigated in HFIM against simulated piperacillin/tazobactam versus meropenem regimens for 0–48 h. ESBL *E. coli* (a) CTAP#168, (b) CTAP#169 and (c) CTAP#173; non-ESBL *E. coli* (d) CTAP#179 and (e) CTAP#180. TZP, piperacillin/tazobactam; MEM, meropenem. Inf, infusion. This figure appears in colour in the online version of *JAC* and in black and white in the print version of *JAC*.

The extent of bacterial killing was >3 log_10_ cfu/mL by 8 h for every piperacillin/tazobactam and meropenem regimens against each ESBL-producing or non-producing *E. coli* (Figure 1).

At the 24 h timepoint, the highest bacterial killing was ∼4 log_10_ cfu/mL for ESBL-producing *E. coli* (CTAP#169, CTAP#173), while ∼4–5 log_10_ cfu/mL bacterial reduction was observed for non-ESBL-producing *E. coli* (CTAP#179, CTAP#180) with both piperacillin/tazobactam regimens. For the piperacillin/tazobactam 6 hourly regimen, the bacterial killing at 24 h was ∼5 log_10_ cfu/mL, and for the 8 hourly regimen, a ∼4 log_10_ cfu/mL bacterial reduction was observed at 24 h for ESBL-producing *E. coli* CTAP#168. For non-ESBL-producing *E. coli* (CTAP#179, CTAP#180), 6 hourly piperacillin/tazobactam attained ∼5 log_10_ cfu/mL bacterial killing while 8 hourly piperacillin/tazobactam regimen achieved ∼4 log_10_ cfu/mL bacterial kill for CTAP#179 at 24 h. Over 72–168 h, piperacillin/tazobactam regimens reduced bacterial load below or at the level of the LLOQ for non-ESBL-producing *E. coli* (CTAP#179, CTAP#180). On the other hand, for ESBL-producing *E. coli* CTAP#169 and CTAP#173, ∼4–5 log_10_ cfu/mL bacterial kill was observed with piperacillin/tazobactam regimens over 168 h (Table S3). Piperacillin/tazobactam administered 6 hourly reduced ∼5 log_10_ of bacterial load while piperacillin/tazobactam administered 8 hourly reduced ∼4 log_10_ cfu/mL over 72–168 h against CTAP#173 (Table S3).

The meropenem regimen on the other hand resulted in ∼5 log_10_ cfu/mL bacterial killing by 24 h (Figure 1), with bacterial counts below the LLOQ for both ESBL-producing and non-producing *E. coli* until Day 7 (Table S3).

#### Effects of piperacillin/tazobactam and meropenem on emergence of resistance

No resistant sub-populations were detected in any piperacillin/tazobactam-treated arms on CAMHA containing piperacillin/tazobactam (32 mg/L of piperacillin, with fixed 4 mg/L tazobactam) for ESBL-producing or non-producing *E. coli*. For untreated control arms of CTAP#168, CTAP#173, CTAP#179 and CTAP#180 *E. coli*, no detectable piperacillin/tazobactam-resistant sub-populations were identified. For the ESBL-producing *E. coli* CTAP#169, the untreated arm had piperacillin/tazobactam-resistant sub-populations (∼2 log_10_ cfu/mL) at the end of experiment. The colonies of these sub-populations recovered at 168 h had an increased piperacillin/tazobactam MIC of 256 mg/L. On the other hand, no detectable meropenem-resistant sub-populations were observed from the meropenem-treated arm or untreated control arm on CAMHA-containing meropenem 16 mg/L for any ESBL-producing or non-producing *E. coli* clinical isolates.

## Discussion

This *in vitro* HFIM study highlights three key findings. Firstly, piperacillin/tazobactam regimens reduced bacterial cells by ∼4–5 log_10_ cfu/mL within 24 h and prevented emergence of resistance over the course of treatment for three ESBL-producing (CTAP#168, CTAP#169 and CTAP#173) and two non-producing (CTAP#179 and CTAP#180) *E. coli* clinical isolates. Secondly, in comparison to 8 hourly meropenem, the 6 hourly piperacillin/tazobactam regimen achieved less killing against one of the three ESBL-producing *E. coli* isolates (CTAP#173) but comparable killing against the other two ESBL-producing isolates (CTAP#168 and CTAP#169) and the two non-producing isolates (CTAP#179 and CTAP#180). Finally, the level of bacterial killing by 6 hourly piperacillin/tazobactam was ∼1 log_10_ greater compared with 8 hourly piperacillin/tazobactam for CTAP#168 and CTAP#179 *E. coli* isolates at 24 h.

There is an increasing interest in the potential efficacy of piperacillin/tazobactam as an alternative to carbapenems, especially for the treatment of urinary infections caused by susceptible ESBL-producing *E. coli*.^35^ Piperacillin/tazobactam in our study decreased bacterial concentrations ∼4–5 log_10_ cfu/mL within 24 h, with no emergence of resistance over the treatment course, which is corroborated with an *in vitro* time–kill study where >3 log_10_ of bacterial killing was observed with piperacillin/tazobactam against urinary ESBL-producing *E. coli*.^36^ Piperacillin/tazobactam antibacterial activity is associated with the duration of the dosing interval for which the free piperacillin/tazobactam concentrations remain above the pathogen’s MIC (ƒ*T*_>MIC_).^37^ In our study, the observed ƒ*T*_>MIC_ was 100% for piperacillin/tazobactam regimens (Table S2). An earlier study suggested that ƒ*T*_>MIC_ of >50%–60% are associated with bactericidal effects.^38^ A later study reported *ƒT*_>MIC_ of 75% threshold was associated with 3 log_10_ bacterial kill.^39^ Further, EMA^40^ and FDA^41^ recommended ∼1–2 log_10_ drops in bacterial cfu as a pharmacodynamic target. Moreover, a reduction in bacterial cfu by ≥2 log_10_ over 24 h, which brings bacterial concentrations below to <5 cfu/g, has been demonstrated in a murine pneumonia model study to accelerate granulocyte-mediated bacterial killing.^42^ Piperacillin/tazobactam regimens in our study reduced bacterial concentrations down to an extent where granulocyte-mediated immune response would likely clear residual bacteria.

Although piperacillin/tazobactam is a potent antibiotic against urinary ESBLs, the presence of genes encoding multiple antibiotic resistance mechanisms may reduce tazobactam’s enzyme inhibition and diminish the efficacy of piperacillin/tazobactam. We observed ∼1 log_10_ cfu/mL lower bacterial killing for piperacillin/tazobactam compared with meropenem against one of three ESBL-producing *E. coli* (CTAP#173). Our result is consistent with a murine sepsis model study, where piperacillin/tazobactam attained ∼1 log_10_ cfu/g lower bacterial reductions compared with imipenem for ESBL-producing *E. coli*.^43^ The lower bacterial killing for piperacillin/tazobactam compared with meropenem, as observed in our study, may be attributable to co-carriage of multiple antibiotic resistance genes in ESBL-producing *E. coli* used in this study (Table 1). The *bla*_CTX-M-15_ ESBL-encoding *E. coli* CTAP#169 co-harboured *bla*_OXA-1_ penicillinase and multiple other resistance genes (Table 1). *E. coli* CTAP#173 carried multiple genes encoding resistance to different antibiotics including *bla*_CTX-M-15_, *bla*_EC_, *bla*_TEM-1_ presented in Table 1. Inhibitor tazobactam exhibits better affinity against common ESBL variants^44^ while weak activity against *bla*_OXA-1_ penicillinase.^22,45^ In this context, the inhibitor–enzyme interaction depends on the number of inhibitor molecules hydrolysed per unit time before an enzyme molecule is completely inhibited, described as turnover number (*t*_n_).^46^ These interactions (*t*_n_) are unique for each β-lactamase.^46^ For instance, tazobactam inhibited PC1 penicillinase (*t*_n _= 2) more rapidly compared with TEM-2 β-lactamase (*t*_n _= 125).^44^ Thus, carriage of multiple β-lactamases including *bla*_CTX-M-15_/*bla*_EC_/*bla*_TEM-1_/*bla*_OXA-1_ observed in our ESBL-producing *E. coli* clinical isolates and their suspected up-regulation could reduce the inhibitory activity of tazobactam,^44^ which may diminish piperacillin/tazobactam efficacy. Further, high bacterial inoculum with pre-existing increased MIC or resistant sub-populations may reduce the effect of piperacillin/tazobactam against ESBL-producing *E. coli*. Nevertheless, bacterial killing observed for the 6 hourly piperacillin/tazobactam regimen in our study was comparable to that observed for the 8 hourly meropenem regimen against two of three ESBL-producing (CTAP#168 and CTAP#169) and the two non-producing (CTAP#179 and CTAP#180) *E. coli* clinical isolates. Overall, the present study supports the utility of piperacillin/tazobactam as a potential alternative to carbapenems for the treatment of urinary sepsis due to susceptible ESBL-producing *E. coli*. However, clinical studies are limited, specifically those investigating the efficacy of piperacillin/tazobactam in the context of ESBL-mediated urosepsis.

A clinical study evaluating the outcome of piperacillin/tazobactam versus a carbapenem in ESBL bloodstream infection did not observe a difference in hospital mortality (3.0% versus 7.8%; *P *= 0.40), hospital length of stays (6.1% versus 5.9%; *P *= 0.88) and ICU length of stay (4.7% versus 3.3%, *P *= 0.39), where 73.0% of bloodstream infection were urinary originated.^47^ Further, piperacillin/tazobactam was recommended in ESBL pyelonephritis as no difference was observed between piperacillin/tazobactam versus carbapenem in terms of clinical resolution or 30 day mortality.^35^ Although the results of MERINO trial^23^ did not support the use of piperacillin/tazobactam against bloodstream infection due to ceftriaxone-resistant *E. coli* or *Klebsiella* spp., that result should not necessarily be generalized to other contexts, particularly in urinary sepsis caused by ESBL-producing *E. coli*. Firstly because the mortality difference was less pronounced with piperacillin/tazobactam when part of the MERINO isolates were retested using reference broth microdilution and after excluding piperacillin/tazobactam-resistant isolates (MIC >16 mg/L, in accordance with EUCAST susceptibility breakpoint for Enterobacterales, 2017).^45^ A significant error was identified with MIC test strips^45^ and thereby MICs may be underestimated in the MERINO trial.^23^ This reflects the importance of using standard and reliable susceptibility testing in clinical microbiology laboratory. Secondly, the mortality difference was relatively lower for urinary versus non-urinary infections (6.9% versus 18.8%).^23^ Thirdly, UTI was more frequent in meropenem compared with piperacillin/tazobactam (67.0% versus 54.8%).^23^ Further, the mortality difference was relatively higher for *Klebsiella pneumoniae* versus *E. coli* (23.1% versus 10.6%), which could overestimate the mortality (12.3%) for piperacillin/tazobactam.^23^ An earlier RCT, focusing on UTI due to ESBL-producing *E. coli*, did not find a statistical difference between piperacillin/tazobactam and ertapenem for clinical or microbiological success rates or mortality at 28 days.^24^ Moreover, data from the Canadian ward surveillance study (CANWARD) surveillance have recently reported 92.7% of ESBL-producing *E. coli* remain susceptible to piperacillin/tazobactam.^11^ Therefore, these data support further clinical investigations for piperacillin/tazobactam as a carbapenem-sparing option for the treatment of urosepsis caused by susceptible ESBL-producing or non-producing *E. coli*.

This HFIM study has some limitations. First, we have used *E. coli* clinical isolates, thus results from our study may not extrapolate to other bacterial isolates. Second, a lack of host immune system with *in vitro* HFIM model, although optimizing bactericidal activity based on optimal dosing regimens simulated in HFIM could predict clinical microbiological outcomes with high accuracy.^48^ Furthermore, our results may best forecast pharmacodynamics of antibiotics in immunocompromised patients, often observed in the ICU. Third, we have considered only one renal function (CL_CR_ of 100 mL/min) when simulating PK profile; however, this represents median baseline CL_CR_ of 103 mL/min observed in patients with complicated UTIs^31^ and is therefore representative of the target population. Fourth, we have selected one or two dilutions (32 mg/L for piperacillin and 16 mg/L for meropenem) above the clinical breakpoint for piperacillin and meropenem to increase the detection of resistant sub-populations. Given the inherent assay variation in the MIC tests,^49^ one or two dilutions above the clinical breakpoint were selected to ensure the detection of resistant sub-populations.

In conclusion, piperacillin/tazobactam (4.0/0.5 g 6 hourly) was comparable to meropenem (1 g 8 hourly) in terms of bacterial killing and prevention of emergence of resistance over the experiment for two of the three ESBL-producing (CTAP#168 and CTAP#169) and also for both of the non-ESBL-producing (CTAP#179 and CTAP#180) *E. coli* clinical isolates. Piperacillin/tazobactam may be a suitable alternative to carbapenems for the treatment of urosepsis caused by most common ESBL (*bla*_CTX-M_)-producing piperacillin/tazobactam-susceptible *E. coli*. Many clinical microbiology laboratories use automated equipment for susceptibility testing; however, the test should be performed by the standard susceptibility testing method. Further, rapid characterization of β-lactamase gene contents in clinical bacterial isolates may prove helpful to determine co-carriage of multiple β-lactamases in susceptible ESBL-producing *E. coli*. A randomized controlled clinical trial with robust design is urgently required to substantiate these results. Further studies characterizing the effects in the presence piperacillin/tazobactam-resistant *E. coli* or biofilm presence are also warranted.

## Supplementary Material

dkac186_Supplementary_Data

## Supplementary data

Supplementary Methods, Tables S1 to S3 and Figures S1 and S2 are available as Supplementary data at *JAC* Online.
