# Supplementary material for: Pharmacodynamic evaluation of piperacillin/tazobactam versus meropenem against extended-spectrum β-lactamase-producing and non-producing Escherichia coli clinical isolates in a hollow-fibre infection model
Source: J Antimicrob Chemother. 2022 Jun 20;77(9):2448–55. doi: 10.1093/jac/dkac186 (PMC9410668; doi:10.1093/jac/dkac186)
Supplement: dkac186_Supplementary_Data [file dkac186_supplementary_data.docx]

**Supplementary data**

**Methods**

***Phenotypic identification of ESBL-producing E. coli***

Production of ESBL was determined phenotypically using combination disk diffusion test.^1^ In brief, three to four *E. coli* colonies of same morphological type were taken from 16 h agar plate to prepare an inoculum of 0.5 McFarland. A sterile cotton swab was dipped into the prepared inoculum and streaked onto CAMHA by Kirby-Bauer method to attain confluent even inoculum. The antibiotic discs were placed onto the surface of the agar plates 3-5 min after inoculation. Disks containing 30 μg of cefotaxime (BD BBL^TM^ Sensi - Disc^TM^, Becton, MD, USA) and ceftazidime (BD BBL^TM^ Sensi - Disc^TM^, Becton, MD, USA), and a combination of either of these two drugs with 10 μg of clavulanic acid were dispensed at minimum distance of 24 mm apart from each other onto the inoculated plate bacterial lawn culture and incubated at 37°C for 16 h. Isolates for which a ≥5 mm diameter increase in a zone of inhibition was observed for either antibiotic in combination with clavulanic acid as compared to the zone diameter observed for the respective antibiotic alone, were considered as ESBL-producers.^1^ The test was conducted in duplicates for each *E. coli* isolate according to CLSI performance standards.^1^ *E. coli* ATCC 25922 and *Klebsiella pneumoniae* ATCC 700603 were used as negative and positive controls respectively.^2^

***Whole genome sequencing and bioinformatic analysis***

*E. coli* clinical isolates were grown on 5% horse blood agar (Edwards Group Pty ltd) and incubated for 24 h at 37˚C. Genomic DNA was extracted from bacterial colonies using the DNeasy^®^ UltraClean^®^ Microbial Kit (QIAGEN) according to the manufacturer’s instructions. The DNA concentration was quantified using a Qubit^™^ High Sensitivity 1x dsDNA assay kit with a Qubit^™^ 4.0 fluorometer (Invitrogen^™^, Thermo Fisher Scientific). DNA libraries were prepared using the Nextera DNA Flex Library Prep Kit (Illumina, Australia) in accordance with the manufacturer’s instructions. The library was assessed by Agilent 4150 TapeStation system using D1000 high sensitivity ScreenTape and reagents. WGS was performed using Illumina MiniSeq, High Output Reagent Cartridge (300 cycles) paired ends according to the manufacturer’s instructions at the University of Queensland Centre for Clinical Research (UQCCR), Herston, Brisbane, Australia.

Genomic analysis was undertaken using a custom, inhouse developed, microbial genomic analysis pipeline (https://github.com/FordeGenomics/SnapperRocks). Firstly, the raw Illumina sequence read data for each isolate was quality trimmed using Trimmomatic (version 0.36)^3^ removing low quality bases and Illumina adapter sequences. Next, using Kraken 2 (version 2.0.7-beta)^4^ a RefSeq database^5^ comprised exclusively of bacterial genomes, sequence read data for each sample was screened for contamination and taxonomic labels were assigned. Quality trimmed sequence reads for each isolate were assembled using SPAdes (version 3.14.1)^6^ with default parameters. Following assembly low quality contigs (contigs length <100bp, coverage <20X) were removed. *In silico* resistance gene profiles were determined by screening the genome assemblies for each isolate against the NCBI resistance gene database^7, 8^ using AMRFinderPlus with default parameters.^7^ Multi-locus sequence typing (MLST) of isolate raw reads and assembled quality filtered contigs was performed using srst2 (version 0.2.0)^9^ and MLST (version 2.16.4) (https://github.com/tseemann/mlst), respectively, and typing schemes available on PubMLST (<https://pubmlst.org>).

***Piperacillin/tazobactam and meropenem assay***

A validated Ultra-High Performance Liquid Chromatography with tandem Mass Spectrometry (UHPLC-MS/MS) method on a Nexera liquid chromatograph connected to a 8030+ triple quadrupole mass spectrometer (Shimadzu, Kyoto, Japan) was used to measure the concentrations of piperacillin and tazobactam in CAMHB. Test samples were assayed in batches along with calibrators and quality controls, and results were subject to pre-established batch acceptance criteria.^10^ A 30 µL aliquot of sample was spiked with 20 μL of internal standard ([d_5_]-piperacillin 25 µg/mL and sulbactam 10 µg/mL) and protein was precipitated using 100 μL acetonitrile. An aliquot of 0.5 μL of supernatant was injected into the UHPLC-MS/MS. The stationary phase was a Shim-pack XR-ODS III 2.0 × 50 mm (1.6 µm) analytical column (Shimadzu, Kyoto, Japan) preceded by a C18 UHPLC analytical guard column (Phenomenex, Torrence, USA). Mobile phase A was 0.1% formic acid in ultrapure water and mobile phase B was 0.1% formic acid in acetonitrile. Mobile phase delivered at a gradient from 10% to 75% of mobile phase B at a flow 0.35 mL/min, producing a backpressure of approximately 9000 psi. Piperacillin and its internal standard [d_5_]-piperacillin were monitored with positive mode electrospray at multiple reaction monitoring (MRM) of 518.0→143.0 and 523.0→148.0, respectively. Tazobactam and its internal standard sulbactam were monitored with negative mode electrospray at MRM of 299.0→138.15 and 232.05→140.05, respectively. Piperacillin was measured over a calibration range of 0.870 to 218 mg/L. The precision was 3.2%, 2.1%, 3.2% and 3.3% at piperacillin concentrations of 2.61, 21.8, 69.6 and 174 mg/L respectively. The accuracy was 0.0, -0.9, -0.2 and 1.2% at piperacillin concentrations of 2.61, 21.8, 69.6 and 174 mg/L, respectively. Tazobactam was measured over a calibration range of 0.109 to 27.2 mg/L. The precision was 4.6, 4.0, 5.9 and 6.4% at tazobactam concentrations of 0.326, 2.72, 8.70 and 21.8 mg/L, respectively. The accuracy was -0.1, -1.0, -3.3 and -0.7% at tazobactam concentrations of 0.326, 2.72, 8.70 and 21.8 mg/L, respectively. The assay method has been adapted from Naicker, S *et al*.^11^ Meropenem in CAMHB were measured by an ultra-high performance liquid chromatography-photo diode array (UHPLC-PDA) method on a Nexera2 liquid chromatograph connected to a SPD-M30 photo diode array detector (Shimadzu, Kyoto, Japan). Test samples were assayed in batches together with calibrators and quality controls and results were subjected to batch acceptance criteria.^10^ An aliquot of 2 μL of the test sample was directly injected onto the UHPLC-PDA instrument. The stationary phase was Xbridge BEH C18 2.1 × 30 mm (2.5 µm) analytical column (Waters, Milford, USA) preceded by a C18 UHPLC analytical guard column (Phenomenex, Torrence, USA). Mobile phase A was 30 mM sodium phosphate in water (pH 3.0) and mobile phase B was sodium phosphate in 50% acetonitrile (pH 3.0). Separations for meropenem was effected by a gradient from 10% to 90% mobile phase B at a flow 0.3 mL/min, producing a backpressure of approximately 3200 psi. Meropenem eluted at 2.2 min and was detected at 304 nm. Meropenem was measured over a calibration range of 0.5 to 100 mg/L. The lower limit of quantitation (LLOQ) was 0.5 mg/L. The precision was within 3.8% and the accuracy was within -6.4% for the meropenem concentrations of 1.5, 25 and 80 mg/L in CAMHB.

**Supplementary tables**

**Table S1.** Sequence data and accession numbers for clinical bacterial isolates

| Sample name | Strain name | Bacteria | Accession | Biosample accession | Infection types | Antimicrobial resistance genes | MLST |
| --- | --- | --- | --- | --- | --- | --- | --- |
| 1-168 | CTAP168 | *E. coli* | SRR15909671 | SAMN21447142 | UTI | *acrF*, *bla*_CTX-M-15_, *bla*_EC_, *emrD*, *mdtM*, *qnrS1* | 2521 |
| 2-169 | CTAP169 | *E. coli* | SRR15909670 | SAMN21447143 | UTI | *aac(3)-IIe*, *aadA5*, *aac(6')-Ib-cr5*, *acrF*, *bla*_CTX-M-15_, *bla*_EC_, *bla*_OXA-1_, *catB3*, *dfrA17, emrD*, *mdtM*, *mph(A)*, *sul1* | 131 |
| 3-173 | CTAP173 | *E. coli* | SRR15909666 | SAMN21447144 | UTI | *aph(3'')-Ib*, *aph(6)-Id*, *acrF*, *bla*_CTX-M-15_, *bla*_EC_, *bla*_TEM-1_, *dfrA14, emrD*, *mdtM*, *mph(A)*, *sul2*, *tet(B)* | 421 |
| 4-179 | CTAP179 | *E. coli* | SRR15909665 | SAMN21447145 | UTI | *aadA2*, *acrF*, *bla*_EC_, *catA1*, *dfrA12, emrD*, *erm(B)*, *mph(A)*, *mdtM*, *qepA4*, *sul1*, *tet(B)* | 38 |
| 5-180 | CTAP180 | *E. coli* | SRR15909664 | SAMN21447146 | UTI | *acrF*, *bla*_EC_, *emrD*, *mdtM* | 38 |

UTI, urinary tract infection, MLST, multilocus sequence typing

**Table S2.** Simulated piperacillin/tazobactam and meropenem dosing regimens, and predicted versus observed pharmacokinetic profiles against *E. coli* clinical isolates in the HFIM

| *E. coli* clinical isolates (MIC) | Dosing regimens | Predicted % *ƒT*_>MIC_ | Observed % *ƒT*_>MIC_ (First dosing interval) | Observed % *ƒT*_>MIC_ (Last dosing interval) |
| --- | --- | --- | --- | --- |
| CTAP#168 (TZP, 4 mg/L; MEM, 0.0156 mg/L) | 4.5 g TZP, every 6 h, 30 min infusion | 100 | 100 | 100 |
|  | 4.5 g TZP, every 8 h, 30 min infusion | 83.8 | 100 | 100 |
|  | 1 g MEM, every 8 h, 30 min infusion | 100 | 100 | 100 |
| CTAP#169 (TZP, 8 mg/L; MEM, 0.0624 mg/L) | 4.5 g TZP, every 6 h, 30 min infusion | 93.8 | 100 | 100 |
|  | 4.5 g TZP, every 8 h, 30 min infusion | 70 | 100 | 100 |
|  | 1 g MEM, every 8 h, 30 min infusion | 100 | 100 | 100 |
| CTAP#173 (TZP, 2 mg/L; MEM, 0.0312 mg/L) | 4.5 g TZP, every 6 h, 30 min infusion | 100 | 100 | 100 |
|  | 4.5 g TZP, every 8 h, 30 min infusion | 97.6 | 100 | 100 |
|  | 1 g MEM, every 8 h, 30 min infusion | 100 | 100 | 100 |
| CTAP#179 (TZP, 4 mg/L; MEM, 0.0156 mg/L) | 4.5 g TZP, every 6 h, 30 min infusion | 100 | 100 | 100 |
|  | 4.5 g TZP, every 8 h, 30 min infusion | 83.8 | 100 | 100 |
|  | 1 g MEM, every 8 h, 30 min infusion | 100 | 100 | 100 |
| CTAP#180 (TZP, 2 mg/L; MEM, 0.0312) | 4.5 g TZP, every 6 h, 30 min infusion | 100 | 100 | 100 |
|  | 4.5 g TZP, every 8 h, 30 min infusion | 97.6 | 100 | 100 |
|  | 1 g MEM, every 8 h, 30 min infusion | 100 | 100 | 100 |

TZP, piperacillin/tazobactam; MEM, meropenem; MIC, minimum inhibitory concentration; % *ƒT*_>MIC_, the percentage of time that free concentrations exceeded the MIC.

**Table S3.** Total bacterial population of *E. coli* clinical isolates investigated in HFIM against simulated piperacillin/tazobactam versus meropenem regimens for 72-168 hours.

| *E. coli* clinical isolates | Time (h) | Total bacterial population (cfu/mL) | | |
| --- | --- | --- | --- | --- |
|  |  | 4.5 g TZP, every 6 h | 4.5 g TZP, every 8 h | 1 g MEM, every 8 h |
| CTAP#168 | 72 | CBL | CBL | CBL |
|  | 96 | CBL | CBL | CBL |
|  | 120 | CBL | 4.70E+02 | CBL |
|  | 144 | CBL | CBL | CBL |
|  | 168 | CBL | CBL | CBL |
| CTAP#169 | 72 | CBL | 1.30E+02 | CBL |
|  | 96 | CBL | CBL | CBL |
|  | 120 | CBL | CBL | CBL |
|  | 144 | CBL | CBL | CBL |
|  | 168 | 3.30E+02 | 4.00E+02 | CBL |
| CTAP#173 | 72 | 3.50E+03 | 6.70E+03 | CBL |
|  | 96 | 4.30E+02 | 5.90E+03 | CBL |
|  | 120 | 3.60E+02 | 3.90E+03 | CBL |
|  | 144 | 2.40E+02 | 2.30E+03 | CBL |
|  | 168 | 1.90E+02 | 5.10E+03 | CBL |
| CTAP#179 | 72 | CBL | CBL | CBL |
|  | 96 | CBL | CBL | CBL |
|  | 120 | CBL | CBL | CBL |
|  | 144 | CBL | CBL | CBL |
|  | 168 | CBL | CBL | CBL |
| TAP#180 | 72 | CBL | CBL | CBL |
|  | 96 | CBL | CBL | CBL |
|  | 120 | CBL | 1.30E+02 | CBL |
|  | 144 | CBL | CBL | CBL |
|  | 168 | CBL | CBL | CBL |

TZP, piperacillin/tazobactam; MEM, meropenem; CBL, counts below LLOQ; LLOQ was 2-log cfu/mL (i.e., counts less than ten colonies per CAMHA were not considered); ESBL-*E. coli* : CTAP#168, CTAP#169, CTAP#173; non-ESBL-*E. coli* : CTAP#179, CTAP#180.

**Supplementary figures**

| 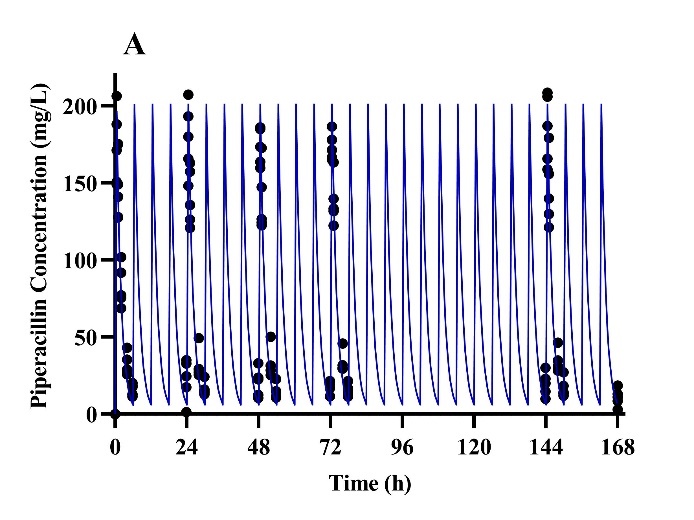 | 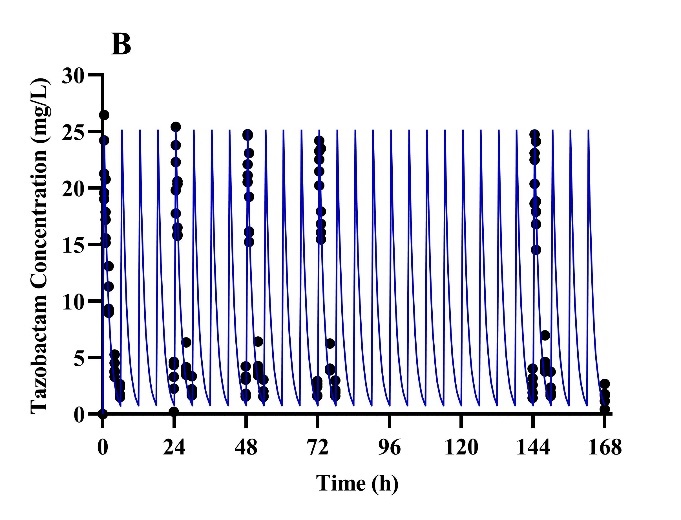 |
| --- | --- |
| 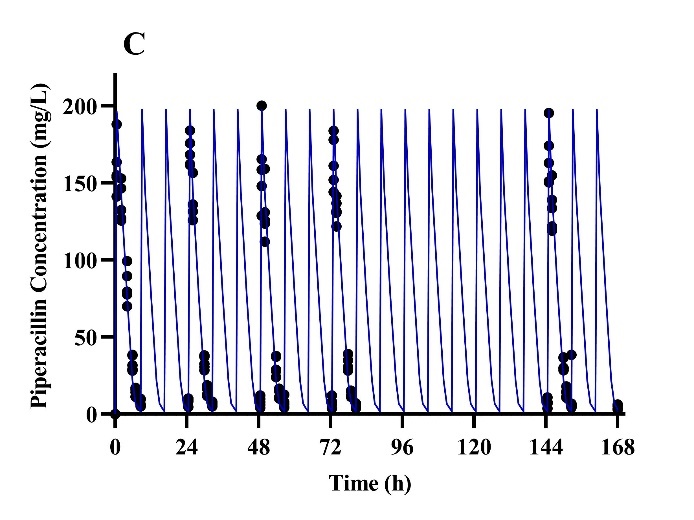 | 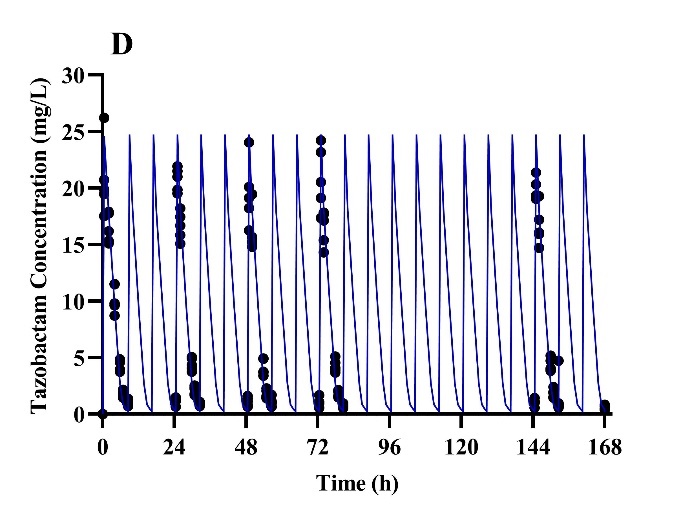 |
| 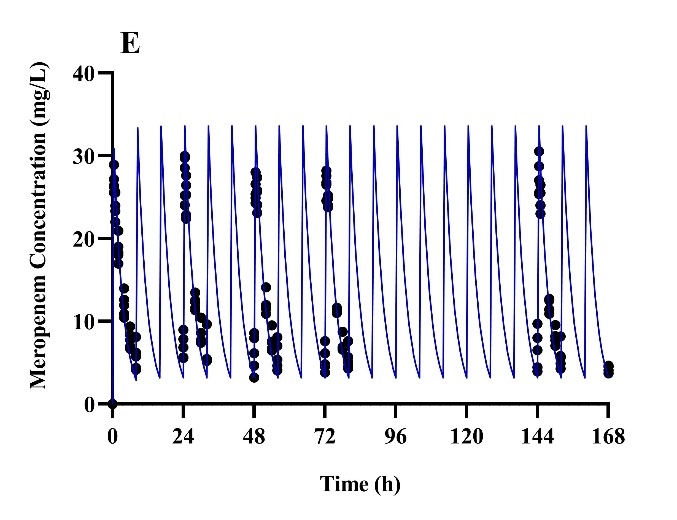 |  |

**Figure S1.** Pharmacokinetic profiles for the HFIM experiments with ESBL-producing and non-producing E. coli isolates. Simulated (solid curves) and observed (filled circles) concentration-time profiles for (A) piperacillin, every 6 h (B) tazobactam, every 6 h (C) piperacillin, every 8 h (D) tazobactam, every 8 h and (E) meropenem, every 8 h. HFIM, hollow-fibre infection model.

| 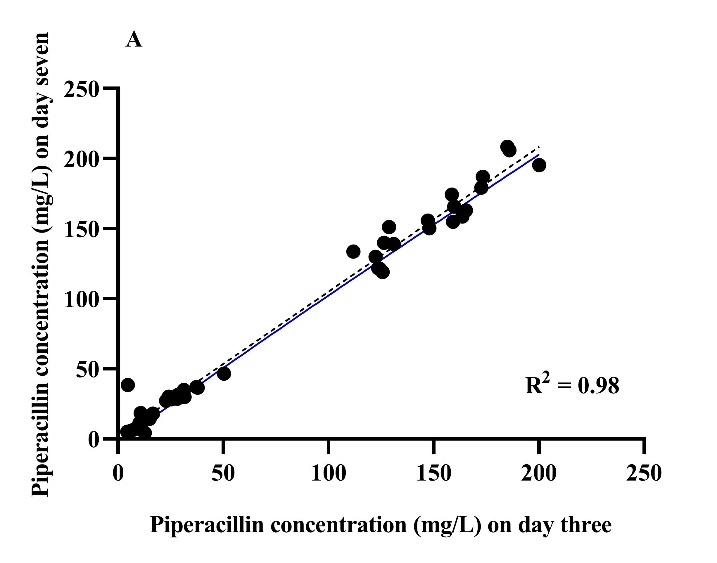 | 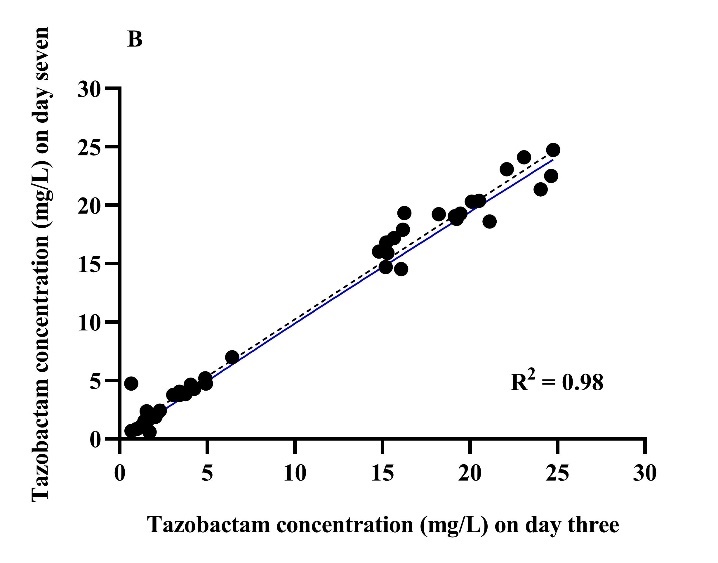 |
| --- | --- |
| 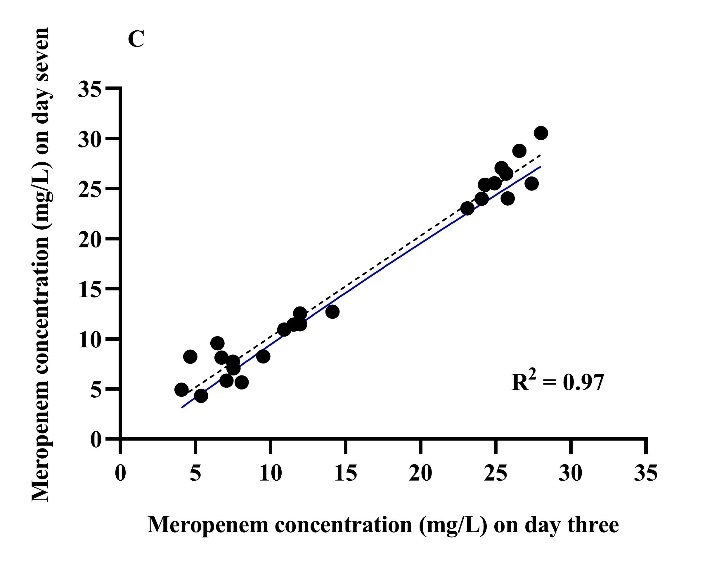 |  |

**Figure S2.** Comparison between a dosing interval for the day three and day seven in the HFIM experiments. (A) piperacillin concentrations (mg/L), (B) tazobactam concentrations (mg/L), and (C) meropenem concentrations (mg/L). HFIM, hollow-fibre infection model.

**References**

1. Clinical and Laboratory Standards Institute. Performance Standards for Antimicrobial Susceptibility Testing. 30th ed. CLSI supplement M100. 2020.

2. Singh N, Pattnaik D, Neogi DK *et al*. Prevalence of ESBL in Escherichia coli isolates among ICU patients in a tertiary care hospital. *J Clin Diagn Res* 2016; **10**: DC19-DC22.

3. Bolger AM, Lohse M, Usadel B. Trimmomatic: a flexible trimmer for Illumina sequence data. *Bioinformatics* 2014; **30**: 2114-20.

4. Wood DE, Lu J, Langmead B. Improved metagenomic analysis with Kraken 2. *Genome Biol* 2019; **20**: 257.

5. O'Leary NA, Wright MW, Brister JR *et al*. Reference sequence (RefSeq) database at NCBI: current status, taxonomic expansion, and functional annotation. *Nucleic Acids Res* 2016; **44**: D733-45.

6. Prjibelski A, Antipov D, Meleshko D *et al*. Using SPAdes De Novo Assembler. *Curr Protoc Bioinformatics* 2020; **70**: e102.

7. Feldgarden M, Brover V, Gonzalez-Escalona N *et al*. AMRFinderPlus and the Reference Gene Catalog facilitate examination of the genomic links among antimicrobial resistance, stress response, and virulence. *Sci Rep* 2021; **11**: 12728.

8. Feldgarden M, Brover V, Haft DH *et al*. Validating the AMRFinder Tool and Resistance Gene Database by Using Antimicrobial Resistance Genotype-Phenotype Correlations in a Collection of Isolates. *Antimicrob Agents Chemother* 2019; **63:** e00483-19.

9. Inouye M, Dashnow H, Raven LA *et al*. SRST2: Rapid genomic surveillance for public health and hospital microbiology labs. *Genome med* 2014; **6**: 90.

10. U.S. Department of Health and Human Services, Food and Drug Administration, Center for Drug Evaluation and Research, Center for Veterinary Medicine. *Bioanalytical Method Validation, Guidance for Industry*, 10001New Hampshire Ave.,Hillandale Bldg., 4th Floor Silver Spring, MD 20993-0002, 2018.

11. Naicker S, Valero YCG, Meija JLO *et al*. A UHPLC–MS/MS method for the simultaneous determination of piperacillin and tazobactam in plasma (total and unbound), urine and renal replacement therapy effluent. *J Pharm Biomed Anal* 2018; **148**: 324-33.
